# Editorial: The impact of shared leadership on group functioning and performance

**DOI:** 10.3389/fpsyg.2023.1125076

**Published:** 2023-03-07

**Authors:** Stewart T. Cotterill

**Affiliations:** School of Rehabilitation, Sport and Psychology, AECC University College, Bournemouth, United Kingdom

**Keywords:** shared leadership, leadership development, athlete leadership, team effectiveness, group effectiveness

Leadership is a fundamental aspect of team functioning across a broad range of performance domains including sport, physical activity, exercise, and health. Indeed, there is increasing recognition that high-quality leadership at all levels within organizations is crucial to the growth and performance of teams. Over the past 25 years, the majority of research has examined leadership from the perspective of the top-down approach that relies on a leader-centric approach. However, this only represents one aspect of leadership within performance domains. Equally important, although far less examined, is the concept of shared leadership within teams, specifically termed “athlete leadership” or “peer leadership.” Leaders within teams have been suggested to have significant impact upon a range of team-related factors including satisfaction, cohesion, team dynamics, performance, health, and well-being. There is limited clarity, however, regarding the best mechanisms through which to develop the leadership capacity and potential within a team.

This Research Topic focuses on exploring shared leadership within a range of different contexts. Specifically seeking to better understand current knowledge, approaches to leadership development, and future avenues for research in the domain of shared leadership. The articles that compose this Research Topic explore a range of perspectives and approaches to better understand shared leadership, the impact that shared leadership can have within a group or team context, and also how to best develop the leaders of the future.

In the first study in the Research Topic Wu and Cormican focus upon exploring both whether shared leadership is positively related to team effectiveness and also when shared leadership is more likely to be effective. The study specifically focuses on achieving this through the use of a social network analysis approach working with a number of Chinese design teams. One of the unique features of this study is that it is among the first to investigate the temporal factors that impact upon the effectiveness of shared leadership.

In the second study Edelmann et al. explore the underpinning mechanisms of the relationships between formal leaders and their team members, specifically focused upon employees from a range of different sized organizations in Belgium. The study offers an interesting insight into the use of empowering leadership styles by formal leaders and the impact these leadership styles have upon work-related factors such as work satisfaction. In addition, the article considers the appropriateness of previously identified leadership styles in sport to a non-sporting/organizational context.

In the next study Butalia et al. consider how predictive captain selection is of leadership quality and leader acceptance. Specifically, the authors explore the views of coaches and athletes from both Flemish football and volleyball teams. The study offers interesting insights into the range of leadership characteristics that might be required in order to positively impact upon leadership quality and leader acceptance in sports teams.

In the fourth study De Backer et al. offer an interesting perspective on whether the behavior of the coach impacts upon, or can be used as a predictor for, the quality of athlete leadership experienced. Building upon organizational justice theory and a social identity approach the study specifically focuses on Belgian volleyball and basketball players and whether the perceived justice of the coach predicts the quality of athlete leadership experienced by the players.

The next study in the Research Topic undertaken by Boisvert et al. evaluates the impact of a leadership development programme with youth ice hockey players. The development programme was designed to assessment the impact of a series of leadership workshops as measured by a range of quantitative and qualitative measures. The study offers interesting conclusions in regard to the importance of leadership education and/or development programme in maintaining levels of desired athlete leadership behaviors.

In the sixth study Toivonen et al. report on the feasibility of a responsibility-based leadership training programme. In this study the authors consider the impact of a leadership training programme that promotes positive youth development, personal and social responsibility, and shared leadership on novice physical activity instructors. The study offers an evaluation of an innovative approach seeking to develop the leadership abilities of novice rather than experienced physical activity instructors in Finland

For the next study Walker and Gould present an evaluation of the National Federation of State High School Association's (NHFS) online captain leader development course. In this study Walker and Gould specifically explore the perceptions of student athletes regarding the training course's effectiveness in improving leadership knowledge and ability. The authors offer some interesting insights into the responses and future research directions.

In the penultimate study of the Research Topic López-Gajardo et al. analyzed the relationship between athletes' perceptions of athlete leadership quality, team identification, inside sacrifice and performance with sports team players across a range of different sports including soccer, beach soccer, basketball and volleyball. The study explores player perceptions utilizing a cross-sectional design survey, offering import and interesting conclusions on relationships between perceived quality of athlete leaders, inside sacrifice, and perceived performance, and between inside sacrifice and perceived performance.

In the final article of the Research Topic Cotterill et al. review the current state of knowledge and understanding relating to leadership and leader development with athlete leader populations. Reviewing contemporary examples and current understanding of approaches to athlete leadership development. Also highlighting future areas for research and applied practice development. Finally, the authors outline that while significant advances in understanding have been made there is still a long way to go, with further clarity required regarding the knowledge, skills and expertise required to undertake the athlete leadership roles in sport, and crucially to better understand how the development of current and future athlete leaders can be maximized.

## Author contributions

This Research Topic summary was written by SC building upon the work submitted as part of the Research Topic.

